# Pre-service teachers' use of ICT to collaborate to complete assessment tasks

**DOI:** 10.1007/s13384-022-00580-x

**Published:** 2022-11-12

**Authors:** Eileen V. Slater, Donna Barwood, Zina Cordery

**Affiliations:** School of Education, Edith Cowan University, 2 Bradford Street, Mount Lawley, WA 6050 Australia

**Keywords:** ICT, Collaboration, Collaborative assessment, Higher education, Pre-service teachers

## Abstract

This research explored the use of ICT products by *n* = 123 pre-service teachers to complete collaborative assessments. Students responded to a questionnaire relating to the use, benefits and limitations, and what would better enable the use of ICT for collaborative assessment purposes. The ICT products favoured by students did not support some key elements necessary for online collaboration, through either student use or product functionality. Poor Internet access was a commonly cited limitation with the effect of reducing access to available ICT skills training. Suggestions for changes to course structure and content and further research are made.

## Introduction

### Collaboration

Regardless of the framework and/or context, collaboration (i.e., teamwork) is consistently represented in the exposition of 21st-century skills (Binkley et al., 2012; Griffin and Care, 2015; Tur & Maŕin, 2015; Voogt & Roblin, 2012). Internationally, collaboration is often referred to as the skills and attributes deemed necessary for successful integration into the 21st-century workforce as a productive member of a knowledge society (Kumar et al., 2012; McKinney & Sen, 2016; Seifert, 2016; Tondeur et al., 2012; Tondeur et al., 2021). A review of over 142,000 job advertisements (Rios et al., 2020), found that the ability to collaborate was the third most described job requirement, present in 22% of job-related criteria, and exceeded only by oral (28%) and written communication skills (23%).

In the researchers’ context of higher education, the university where the researchers are employed, positions collaboration as a social process, “necessary for successful integration in the workplace” (Edith Cowan University, 2021) and a key component of global citizenship; developing futures-oriented graduates who are fit for employment (Edith Cowan University, 2018). In the specific context of Initial Teacher Education (ITE) and ITE Institutions (ITEI) in Australia, pre-service teachers are prepared during their undergraduate course to be members of teams, with skills to work collaboratively with their colleagues to meet professional standards for Australian teachers (AITSL, 2017) and subsequently, registration as graduate teachers. Beyond this, the three-dimensional design of the Australian Curriculum informing Australian schooling defines discipline knowledge, cross curricular priorities, and a set of general capabilities, described as “playing a significant role" within the curriculum, “equipping young Australians to live and work successfully in the twenty-first century” (ACARA a, no date, general capabilities, para. 1). These capabilities include “personal and social capability” (ACARA, 2020), which encompasses the ability to effectively collaborate with others. Therefore, in Australia, it is imperative that graduate teachers enter the workforce, not only with the ability to collaborate themselves but with the capacity to develop collaborative skills and dispositions in the students that they teach (ACARA, 2020).

### Collaboration in higher education

Vygotsky's sociocultural theory (1987) can inform collaborative learning activity in higher education settings (Barkley et al., 2014; Erbil, 2020; McKinney & Sen, 2016). For an activity to be considered collaborative learning in this context, students predominantly need to apply joint effort to achieve shared learning outcomes (Laal & Laal, 2012). This includes students working together to complete group assessment tasks, also called collaborative assessments.

Research shows that there are a number of academic (Barkley et al., 2014; Tomacho & Foels, 2012), well‐being and retention (Loes, et al., 2017) benefits attributed to collaborative learning in higher education, as long as the principles of best practice are applied. The limitations of collaborative learning include the negative perceptions that some students have towards collaborative assessment. This includes but is not limited to issues arising from ‘social loafing’ or benefitting from the efforts of others (Strijbos, 2016) and differences in students’ abilities (Roberts & McInnerney, 2007; Van Aalst, 2013). Some studies have proposed solutions to the limitations of collaborative learning, including collaborative assessment (Le, et al., 2018; Roberts & McInnerney, 2007; Van Aalst, 2013) and such learning experience will remain important preparation to enable future workforce readiness.

### Collaboration through ICT in higher education

The International ICT (Information Communication Technologies) Literacy Panel (2002), defined “ICT Literacy” as “using digital technology, communications tools, and/or networks to access, manage, integrate, evaluate, and create information in order to function in a knowledge society” (p. 2). Mac Callum et al., (2014) noted that the term is used as a “measure of an individual's ability to use digital technology” (p. 9). In Australia, pre-service teachers graduate with ICT literacy which is measured according to the professional standards for teachers and specifically, Standards 2.6, 3.4 and 4.5 [Australian Institute for Teaching and School Leadership (AITSL), 2017].

Irrespective of the immediacy of the COVID pandemic (World Health Organization (WHO), 2020) gripping the world, interest in the tools and methodologies supporting digital collaborative learning in the online learning environment in the higher education sector is gaining momentum (Bond et al., 2018; Foulger et al, 2019; McKinney & Sen, 2016). Mannisto et al. (2020) define collaborative learning in a digital context as “a goal‐oriented activity of a group of students committed to achieving a common goal and interactively creating new knowledge in digital learning environments” (p. 2). Bond et al. (2018) describe digital collaboration as a crucial graduate attribute whilst McKinney and Sen (2016) acknowledge the skill set required as rich and complex. Cloud-based tools are noted to have particular potential in supporting digital collaborative learning through the facilitation of “commenting, presenting ideas, sharing screen, chatting and sending instant messages, uploading files, drawing, downloading, storing, and posting.” (Al-Samarraie & Saeed, 2018, p. 89). These tools are often accessed by students due to synchronous or asynchronous functionalities, with immediacy favoured for reducing the disruptions afforded through delayed response time (McKinney & Sen, 2016).

Al-Samarraie and Saeed (2018) conducted a systematic review of the cloud-based technologies used to support digital collaborative learning; reporting results for synchronised tools, Learning Management System (LMS) tools, and social networking tools (p. 79). Their review reported that cloud-based technological tools are predominantly used by students for editing and collegiate discourse. They evidence Google Docs as stimulating student reflection, increasing student motivation, improving cognitive learning strategies, improving self-efficacy, and increasing the sharing of resources. Google Drive was noted as having organisation and sharing benefits, along with increasing student capacity to critique knowledge through iterative feedback processes.

LMS tools (specifically Blackboard as the relevant platform utilised in the research context) were reported as beneficial for tracking individual contributions to collaborative assessment, student motivation to engage in learning, and students’ sense of connectedness and efficacy toward that learning (Al-Samarraie & Saeed, 2018). Social networking was positioned for its potential to expand learning beyond the confines of the educative learning space(s).

Reflective of international research (Au Yond & Yeoh, 2015; Martini & Cinque, 2011; Siefert, 2016), Al-Samarraie and Saeed (2018) detailed the strengths of social networking tools in on-line collaboration to promote higher levels of engagement, team rapport building, remote synchronous discussion, improved self-esteem, and improved performance. However, to maximise the benefits of digital collaborative learning, they advocate that students need to be proficient in the ICT tool/s being used. Like Martini and Cinque (2011) and Kumar et al. (2012), they consider skill as a limiting factor in digital collaborative learning, along with compatibility issues between some ICT tools. This is in the same way that the adoption and uptake of technologies in higher education learning is dependent on the skill, accessibility, and capacity of the institution and those that are charged with its use (Foulger et al., 2019; Kay, 2006).

Lounsbury et al. (2015) in their exploratory research in ITE found that: “Higher education students’ use of technologies has been documented over the years but their specific use of technologies for assessment-related tasks has yet to be fully investigated” (p. 202). They document the perceived benefits and limitations of ICT products and possible inhibitors of ICT in the completion of collaborative assessment tasks. The purpose of their research was to inform future course design relating to ICT product preferences, skill requirements, and availability, and to further enable digital collaboration in assessment.

In light of Lounsbury et al.’s (2015) research and further developments including the COVID 19 pandemic, the research reported here explored pre-service teachers at an ITEI in Western Australia (WA) as digital collaborators. The research specifically investigated the technologies that pre-service teachers used to collaborate when completing assessment tasks. Note that the tasks were collaborative assessment tasks, inclusive of but not limited to *online* collaborative assessment task. Although the use of technology in higher education is supported by policy and research, the researchers found that collaborative assessment completion in a digital space has not received focussed attention. We acknowledge other research such as McKinney and Sen (2016) but position Lounsbury et al. as the only comparable research undertaken internationally, and advocate for further research in this space. We believe the paper will be of value to those working with students in ITE and higher education contexts because of the insights shared on what ICT participants are choosing to engage with, how they view that engagement (benefits and limitations) and how further engagement can be supported.

## Research questions

Using Lounsbury et al.’s research vision as insight, the following research questions informed the basis of the paper:

1. What ICT are pre-service teachers using to collaborate on university assessment tasks?
2. What do pre-service teachers perceive as the benefits and limitations of ICT for the purpose of completing collaborative assessment tasks?
3. What do pre-service teachers perceive would enable the use of ICT for the completion of collaborative assessment tasks?

## Methodology

A convergent, parallel design was used to explore the research questions. Cross-sectional survey research methodology (Creswell, 2014) was used to gather information in relation to students’ current use and perceptions of ICT for the purpose of collaborating with their peers to complete ITE assessments. A cross-sectional survey design is frequently used to explore current “attitudes, beliefs, opinions or practices” (Creswell, 2014, p. 403). This survey was pragmatic in its collection of both quantitative information regarding practice and qualitative data to explore beliefs and opinions. The data could then be analysed to answer individual research questions and also compared for consistency. In the absence of a detailed description of a phenomenon, the theorising of relationships lacks informed predictive grounds. The methods employed allowed the researchers to explore the contextualised participants’ viewpoints and thoughts to inform the necessity for any type of further research that may be required (Crowe et al., 2015).

### Methods

A web-based questionnaire was developed which consisted of demographic information, eight multiple-choice items, and eight open-ended response format items. The questionnaire was promoted during a core unit in the fourth year (final year) of the undergraduate secondary teacher education programme at the ITEI in WA. Barwood et al. (2020) reported that similar units are completed by secondary education pre-service teachers in Australia, regardless of their teaching discipline (learning area). The delivery mode for this unit offers students the opportunity to collaborate during on-campus learning activities and online. A third party (not the researchers) promoted the questionnaire, highlighting the purpose and the opt-in nature of the research and negating any ethical issues arising due to the unequal relationship between the researchers and the students. A follow-up email with a link to the questionnaire was sent to students following the initial promotion and again, by the third party. The questionnaire was made available for four weeks.

### Sample

After data were cleaned, *n* = 123 final year secondary education pre-service teachers, from a total population of 205, formed the sample. This represented 60% of the total population. At the 95% confidence level, the confidence interval is 0.056 and the relative standard error is 5.74%. Table 1 summarises the characteristics of the sample in terms of gender, age, and major teaching area. The percentages are also representative of the broader final year secondary cohort for the gender, age, and course major variables.

**Table 1:** Composition of the sample

Factor	Frequency	Percent
**Gender**		
Male	46	37.4
Female	77	62.6
**Age**		
20 or below	5	4.1
21–25	75	61.0
26–30	22	17.9
31–35	10	8.1
36–40	7	5.7
41–45	2	1.6
51–55	2	1.6
**Major teaching area (discipline)**		
Science (biological)	5	4.1
Design and technology	14	11.4
Drama	7	5.7
English	12	9.8
Health and physical education	39	31.7
Home economics	12	9.8
Humanities and social sciences	10	8.1
Mathematics	9	7.3
Music	4	3.3
Visual arts	11	8.9

### Data analysis

The frequency of use of various ICT was calculated for all responses and the total cases. Open response items were inductively coded to thematically locate critical information through a process of constant comparison (Xu & Zammit, 2020). This involved an independent full reading of the responses by two researchers, followed by the development of codes for similar text sequences on repeated readings, which were subsequently represented as codes. Responses were compared between researchers’ initial code books and comparisons between the two sets of data codes. Codes were subsequently discussed, added to, combined, and refined until agreement was reached. Codes were then grouped into similar themes. In total, data were analysed four times:

1. Independent thematic coding by researcher one,
2. Independent thematic coding by researcher two,
3. Combined constant comparison and
4. Code agreement and refinement.

## Results

### Quantitative results

Table 2 summarises the Microsoft (MS) products that students reported using to complete university collaborative assignments, ranked by percentage of responses. MS Word and MS PowerPoint were the most frequently used products, with approximately 92% of the sample indicating that they used MS Word. A small percentage of students reported using other MS products, including Excel and Prezi.

**Table 2:** Microsoft ICT used for collaborative assessment: frequencies

ICT	Responses		Cases (*n*-123)
	*n*	Percent	Percent
Forms	4	1.3	3.3
Other	7	2.2	5.7
No Microsoft products used for this purpose	7	2.2	5.7
Teams	21	6.7	17.1
One note	23	7.3	18.7
One drive	40	12.8	32.5
PowerPoint	98	31.3	79.7
Word	113	36.1	91.9
Total	313	100.0	

The Google ICT products used to complete collaborative assessments are presented in Table 3. Google Docs was the most widely used Google ICT product, while almost one-third of participants had never used a Google product to support their collaborative assessment efforts.

**Table 3:** Google ICT used for collaborative assessment: frequencies

ICT	Responses		Cases (*n* = 123)
	*n*	Percent	Percent
Hangouts	2	1.1	1.7
Other	5	2.6	4.2
Site	10	5.3	8.3
Forms	14	7.4	11.7
Sheets	17	8.9	14.2
Slides	24	12.6	20
No Google products used for this purpose	38	20	31.7
Docs	80	42.1	66.7
Total	190	100.0	

At the time of writing, Blackboard is the LMS utilised by the ITEI that the sample of pre-service teachers attends. As indicated in Table 4, slightly over half of the sample used the Blackboard Discussion Boards and Blackboard Collaborate to facilitate the completion of collaborative assessments. Blackboard Collaborate is a communicative conferencing function within the Blackboard LMS that allows students (pre-service teachers) and academic staff to converse and interact remotely through online virtual conferences. Blackboard Collaborate can record and store the online meetings and is available to support lecturer-to-student conferences or student-to-student conferences. As with Google products, approximately one-third of participants had not used any Blackboard tools to collaborate with their peers for assessment purposes (Table 5).

**Table 4:** Blackboard ICT used for collaborate assessment: frequencies

ICT	Responses		Cases (*n* = 123)
	*n*	Percent	Percent
Other	2	0.9	1.6
Wiki	8	3.5	6.5
Journals	10	4.4	8.1
Groups	29	12.8	23.6
No blackboard products used for this purpose	43	19	35
Discussion board	64	28.3	52
Collaborate	70	31	56.9
Total	226	100.0

**Table 5:** Social media ICT used for collaborative assessment: frequencies

ICT	Responses		Cases (*n* = 123)
	*n*	Percent	Percent
Viber	2	0.9	1.6
Twitter	6	2.8	4.9
Other	5	2.4	4.0
No social media platforms used for this purpose	9	4.2	7.3
WhatsApp	18	8.5	14.6
Snapchat	29	13.7	23.6
Instagram	30	14.2	24.4
Facebook	113	53.3	91.9
Total	212	100.0	

The most utilised social media platform was Facebook, representing over half of the responses for social media ICT and utilised by 91.9% of participants. Facebook, as an ICT product, included all aspects of the platform, including Facebook Messenger. The other category in social media ICT included Discord.

**Fig. 1:**
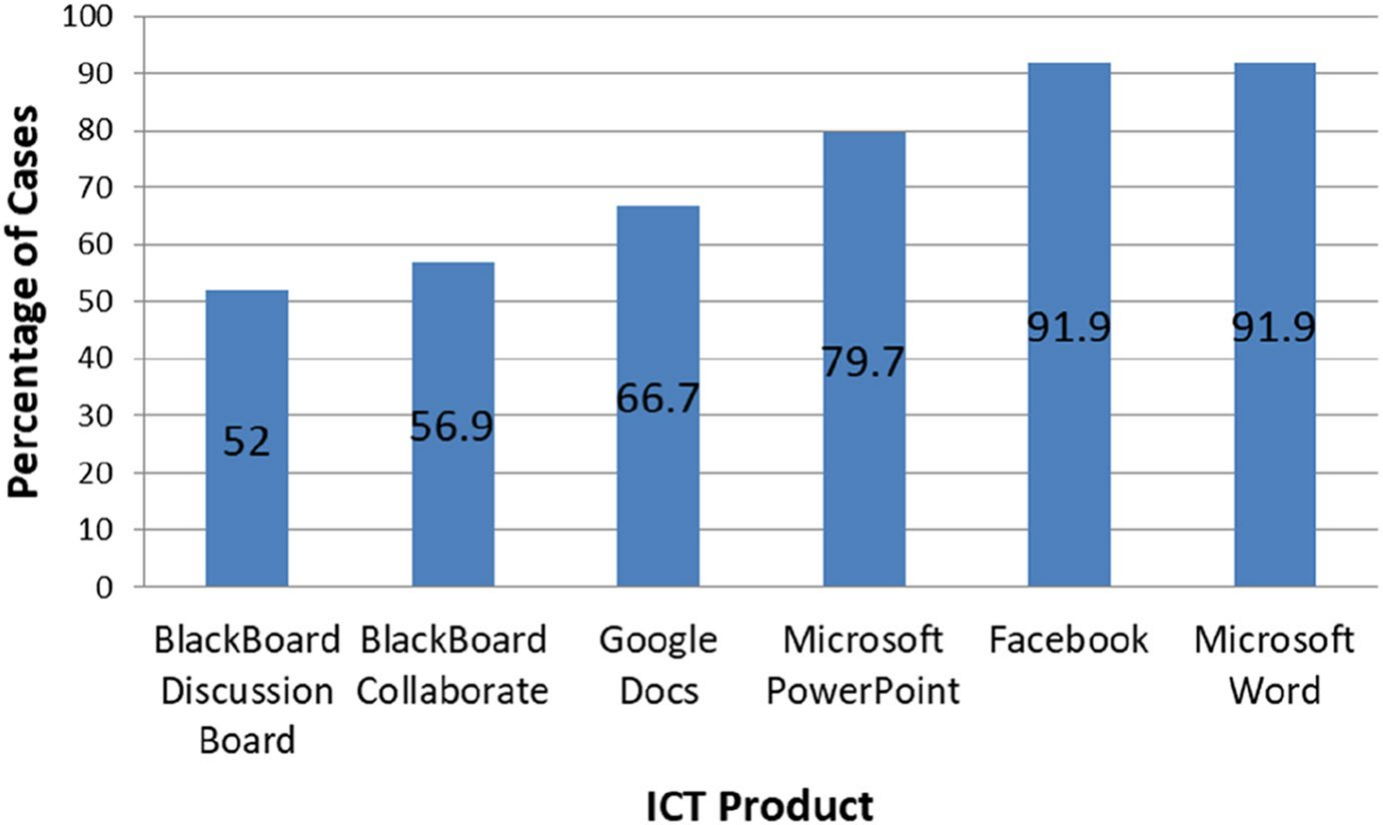
ICT with over 50% reported usage to facilitate collaborative assessments (*n* = 123)

Overall, Microsoft ICT products were the most frequently used ICT products, by total responses, while Google ICT products were the least frequently used. On a product-by-product basis (Fig. 1), the highest cited usage for collaboration on assessments was for MS Word and Facebook, both recording use by 91.9% of cases.

### Qualitative results

Eighty-nine written responses described the perceived benefits of using ICT to collaborate with peers when completing university assessments at the ITEI. These are summarised in Table 6. Four broad themes emerged: accountability, organisation and storage, enhanced communication, and ease of use. The theme of accountability encompassed the students’ ability to track individual contributions to the group assessment and monitor group progress. Organisation and storage referred to the ability to store and view multiple types of files (jpg, mp4,.doc,.ppt etc.) in a single space and the ability to store and retrieve previous versions of work. Enhanced communication encompassed the capacity to work together over time and physical distance, the speed of communication and synchronous document editing, including commenting on changes as they were being made. Ease of use included ease of access, ease due to familiarity, uniformity of formatting and file sharing (Tables 7, 8).

**Table 6:** Benefits of ICT for collaborative assessment

Theme	Example	Percent (*n* = 89)^a^
Accountability	“Google Docs is fantastic as you can see live how much contribution and progress each group member has made.” (ID118)	9
Organisation and storage	“…kept everything in one place” (ID91)	3
Enhanced communication	Google docs and slides made it easy to work simultaneously with other group members. (ID119)	43
Ease of use	Simple to use for most people, something most people already have. (ID94)	48

^a^Responses may have referred to more than one benefit or product

**Table 7:** Limitations of ICT for collaborative assessment

Theme	Example	Percent (*n* = 38)^a^
Access	“Internet Access” (ID19)	50
Skill	“Unsure of how to 100% use the products.” (ID32)	21
Transferring Files	“Sometimes the file size is an issue…” (ID111)	13
Formatting	“I had some formatting issues with Microsoft word and PowerPoint.” (ID45)	11
Compatibility	“I found drop box difficult to use as it was not compatible with my device.”	5

^a^Responses may have referred to more than one benefit or product

**Table 8:** Benefits and limitations by product (count)

	Forms	Teams	One note	One drive	MS power point	MS word	Hangouts	Site	Forms	Sheets	Slides	Docs	Wiki	Journals	Groups	Discussion board	Collaborate	Viber	Twitter	WhatsApp	Snapchat	Instagram	Facebook	Other
**Benefits**																								
Accountability											1	7												
Organ. and storage		3																						
Enhanced Coms											2	26											10	
Ease of use					2	5						12											24	
**Limitations**																								
Access					6	12															2			
Skill				2													6							
Transfer. files					1	4																		
Formatting					1	3																		
Compatibility																	1							1

Thirty-eight written responses described the perceived limitations of using ICT to collaborate with peers to complete university assessments. From these responses, five broad themes emerged: access, skill, transferring files, formatting, and compatibility. Access encompassed Internet issues, hardware access, licensing, limited access to previous work, and limited access due to grouping arrangements within the software. Students’ expressions of limitations in their ability to use programmes were coded under the theme ‘skill’. Transferring files involved limitations in the ability to transfer large files and the need to transfer files to collaborate. The final two themes related to changes in formatting between programmes and programmes being incompatible with hardware.

Students were asked to provide feedback on both awareness and or training, and tool availability or improved functionality, which would support their use of ICT to complete collaborative assessments. Eight themes emerged from the students’ comments (*n* = 60) regarding awareness and or training and four themes emerged concerning availability and functionality. The results are presented in Table 9. One-fifth of the responding students commented that the university already had sufficient product awareness and training in place for students. The most requested training types were ‘online anytime support’ and ‘face-to-face support’, both mentioned in 16% of comments. This result is discussed in relation to currently available supports in the discussion. Some respondents felt a very specific approach “one system to follow” (ID 88) was necessary while others wanted a broad approach.

**Table 9:** University support for students to use ICT for collaborative assessment

Theme	Example	Percent
Awareness or training (*n* = 42)		
Online anytime support	"… video of someone who is experienced in using the program" (ID96)	16
Face to face support	“Mini workshops or drop in sessions for assistance with online collaboration tools could be beneficial.” (ID4)	16
Early intervention	“The ICT unit being moved to first year will be a very big help for future students.” (ID5)	16
Industry focus	“OneNote is being increasingly used in schools, some basic training could help shift people to that.” (ID104)	9
Systematic approach	“There needs to be consistency across the board, different tutors, lecturers, subject areas and departments use different programmes. Would be efficiency [sic] if there was one system for all to follow.” (ID88)	4
Broader approach	“The university could show more of a wider range of collaborative assessment work. The university tends to rely heavily on Word and PowerPoint as opposed to Google docs and other programmes to share knowledge and resources.” (ID89)	4
Awareness through classes	“Day/Week 1 of unit run through valid ICT that could be used in the unit.” (ID47)	14
Already sufficient	“I feel the university offers a good range of ICT assistance already.” (ID21)	21
Tools or functionality (*n* = 42)		
Hardware	“I would absolutely love individual computer rooms so that I could engage in conference calls without disturbing those in the library.” (ID121)	7
Specific software requests	“Zoom” (ID118)	5
Teacher specific ICT	“[Software] that it is easy to design and create student worksheets through.” (ID115)	5
Enable blackboard groups	“Group selections on BB possibly. With grouped folders to allow students to upload/access content during collaborative assessments.” (ID21)	12

## Discussion

### What ICT are students using to enable collaboration on university assessment tasks?

The participants cited Facebook (91.9%) and MS Word (91.9%) as the most prevalent tools used, with PowerPoint also utilised by 80% of participants. The use of Facebook, through the Facebook platform or the Messenger platform, allows for collaboration on assessments through students working synchronously or asynchronously (Cerdà & Planas, 2011). Files and resources can be shared through the platforms, together with communication of their progress (Awidi et al., 2019). The use of social media platforms, like Facebook and Twitter, by students to support their assessment tasks is not something that is actively encouraged by the university, preferring the use of the university-provided IT tools like Blackboard Social Learning tools and PebblePad (Edith Cowan University, 2022). The university policy regarding social media use acknowledges that staff and students may choose to utilise these platforms for sharing and collaborating but suggests that the permanency of the interactions on these platforms is something to consider when using them (Edith Cowan University, 2022). Both MS Word and MS PowerPoint can support digital collaborative learning due to the synchronous editing and commenting available via sharing to OneDrive. Utilising synchronised editing and iterative discourse increases the likelihood of cognitive dissonance and learning (Barkley et al., 2014). The nature of the data did not allow the researchers to understand the extent to which the full functionality of MS Word, MS PowerPoint and Facebook were being utilised. For example, it cannot be determined if MS Word was being used via OneDrive or being moved between participants via e-mail, which would reduce the possible benefits of using the program for collaborative assessments. However, given that only one-third of the respondents were using OneDrive, and that students listed file transfer and formatting as limitations, it is unlikely that the full functionality of MS Word and MS PowerPoint for synchronised editing and discourse was being utilised by most.

Under the theme a ‘broader approach’, students independently drew attention to tertiary staff primarily using MS PowerPoint and MS Word in the facilitation of learning. These data highlight that it is possible that the use of these two ICT tools is reflective of what is being modelled and or embedded in assessment structures, rather than reflective of student preference or skill (Bond et al., 2018). It is advisable that the ITEI and particularly the school to which the participants are enrolled, conduct an audit to ascertain what ICT tools are being embedded into units and specifically into assessment tasks. This would also offer a starting point to address some of the other themes perceived by students as necessary to support the use of ICT to complete collaborative assessment tasks. Like Bond et al. (2018), we also advocate professional learning to support the digital literacy of academic staff.

### What do students perceive as the benefits and limitations of ICT for the purpose of completing collaborative assessment tasks?

Students’ responses show that they value: ease of use, organisation and storage, enhanced communication, and accountability as benefits of the use of ICT to complete collaborative assessments. Facebook, MS Word and MS PowerPoint were the most frequently used tools. The perceived benefits of MS Word and MS PowerPoint were restricted to ease of access, and ease of use due to familiarity; conversely, the benefits of Facebook included ease of access, ease of use due to familiarity, ease of file sharing and speed of communication. Facebook is the most utilised social media networking site in the world, with the company self-reporting 1.13 billion daily users (2016). In Australia, Lord (2016) reported that 93% of social network site users were using Facebook. It is therefore not surprising that students find it easy to use and access. Sadowski et al. (2017) reported that 86% of the students at one Australian university reported having a Facebook account and 80% reported it was their most frequently used social networking site, with 76% of those students checking their account daily. These usage patterns support the speed of communication reported as an advantage by students in this study, noting how quickly peers responded to communication via Facebook.

While used by fewer students, Google Docs was considered to have the most diverse benefits, this included all the elements of ease of use, enhanced communication, and accountability. The benefits of enhanced communication, such as synchronous editing and commenting were cited by Barkley et al. (2014) as essential in ensuring that the use of ICT for collaborative purposes maintains the elements necessary for successful collaboration which leads to learning.

There are implications for the contrast between the tools chosen for use and the ones which reflect the most perceived benefit. One in three, fourth-year students had never used Google Docs and two in three had never used OneDrive, and therefore not used the full functionality of either MS Word or MS PowerPoint. The successful use of ICT to support learning requires students to have good time management skills, discipline, computer skills and opportunity for timely and responsive student–student interaction (Barkley et al., 2014; Sadeghi, 2018). Ensuring that students have access to, familiarity with and the skills to utilise tools which better enable these core elements through embedding use throughout the four years of the undergraduate degree course is desirable.

As reported by Al-Sammarraie and Saeed (2018) student skill level and compatibility were limitations of ICT when completing collaborative assessments, however, 50% of students reported Internet access or Internet speed as the most limiting factor in this study. While the researchers were aware that Australia’s Internet speed is rated 50th in the world (Thompson et al., 2017), they were unprepared for the degree to which Internet issues were hindering students’ ability to use ICT for completing collaborative assessments. The university offers free Internet access to students while on campus, however, ICT is used to collaborate over distance and time, an advantage noted by participants. Therefore, coming onto campus to collaborate detracts from a key benefit of using ICT for collaborative purposes. Oyedemi (2012) found that university students in South Africa had differential access to the Internet beyond university campuses and other open access spaces. Poor access was found to affect students unequally, with race and geographic region cited as key determinants. Research into the pre-tertiary education environment in Australia found similar equity issues (Ma, et al., 2019; Thomas et al., 2020). Research needs to be conducted in the researcher's context to determine if the same equity issues are prevalent, particularly given the diverse nature of the student population in terms of socio-economic status, English second language status, and cultural diversity. As stated by Arquero et al. (2017), it is important for higher education institutions to better understand student motivation and adoption of technology use, to support success when completing assessments through ICT enabled collaboration.

### What do students perceive would further enable the use of ICT for the completion of collaborative assessment tasks?

While 21% of students who responded to these questions reported that the university currently offered sufficient support in the use of various ICT products, by way of agency and access, a total of 32% of students wanted more training, either in person or online. This may be the result of a lack of awareness among students of when, where and how to access support. The ITEI currently offers free access to a suite of software products including MS Office and MS Teams. In addition, online anytime support is offered through LinkedIn Learning. At the time of writing, for example, there were 56 ‘courses’ available through LinkedIn learning for MS Word, ranging from 31 min to 13 h in length. Students have access to the Linkedin learning courses through a direct link on their portal home page. In addition to this, each Blackboard site has an embedded Student Support Services link, which among other things, facilitates access to the Academic Skills Centre, which offers courses in End Note, Panopto and PebblePad. Further to this, the students can access peer support through the libraries ‘Peer Support with Learning Technologies’ program, accessible through MS Teams. We note that some of this support is offered in online learning spaces, and as students reported limitations to their Internet access and speed, this would impact their capacity to engage in some of the available support.

A number of students (14%) suggested awareness raising in relation to the availability of software and training should occur at the commencement of each unit. Further to this, 16% of students found the core ICT unit very useful in raising both their awareness of and level of skill in using various software programmes, suggesting the unit be moved into the first year of the course. These elements combined with an expansion of the use of Google Docs and OneNote, to ensure teachers are prepared to meet the reported expectations of their industry and that students are not limited to MS Word and MS PowerPoint, are all possible modifications for the faculty at the university to consider.

## Conclusion

With the increased use of collaborative assessments and the movement toward online modalities, universities need to consider the access to, and skills in the use of ICT required by students for collaborative assessment purposes. Neither access nor skills can be assumed. It is evident from this sample that some ICT products are not being used in a manner that supports the development of collaborative skills, even when they have the facility to do so. The results elicit questions surrounding the types of collaborative tasks being set by the faculty, the necessity to collaborate *online* to complete those tasks, and how ICT products are explicitly being used to support the completion of collaborative assessments; are the means truly collaborative at all? Further research into these areas will enable a focussed approach to when and how students are asked to complete collaborative assessments and which ICT products should be embedded into those tasks to maximise the benefits of the collaborative process and increase student awareness and skill in using a wider variety of ICT products.

In relation to access, this research has highlighted a substantial access problem for higher education students in this sample which will require further research with a sense of immediacy, and subsequent solutions to avoid potential equity issues.

It can also be concluded that faculty are heavily reliant on MS PowerPoint and MS Word and it is advisable to update their skills in relation to the ICT products which best support collaborative learning in online environments and those required by the students’ potential employers.

## Limitations

While the sample is large enough to be generalised to the broader student body at the university, generalisation beyond that would depend upon multiple contextual variables. Further cross-institutional studies would be beneficial, including student access to the Internet as a limiting factor.

While the researchers asked for the benefits and limitations of each ICT tool in completing collaborative assessments, the data did not specify exactly how each ICT product was being used. The researchers acknowledge the need to follow up on the initial exploration to collect data on the very specific implementation of each ICT tool in collaborative assessment.
